# Assessing the Best Supplier Selection Criteria in Supply Chain Management During the COVID-19 Pandemic

**DOI:** 10.3389/fpsyg.2021.804954

**Published:** 2022-04-29

**Authors:** Yumei Hou, Maryam Khokhar, Sayma Zia, Anshuman Sharma

**Affiliations:** ^1^School Of Economics and Management, Yanshan University, Qinhuangdao, China; ^2^School of Management, Yangen University, Quanzhou, China; ^3^Department of Business Studies, Bahria Business School, Bahria University Karachi Campus, Karachi, Pakistan; ^4^Department of Marketing, College of Business Administration, Ajman University, Ajman, United Arab Emirates

**Keywords:** supply chain management, supplier selection, environmental performance, fuzzy TOPSIS, social interests, COVID-19

## Abstract

In the last 10 years, organizations and researchers have recognized the importance of sustainable supply chain management (SSCM) because of the consumers, -profit and non-profit organizations, laws and regulations, and consumer social and corporate responsibilities. Supplier selection, environmental effects such as social cooperation, and other SSCM programmes, can all help to achieve the “triple bottom line (TBL)” of economic, environmental, and social advantages. Sustainable supplier selection (SSS) and firm performance are important factors in supply chain management (SCM). Organizations will traditionally consider a new framework when evaluating SSS performance to obtain all-encompassing criteria/sub-criteria of the sustainability index by encapsulating sustainability. This paper compiles 12 subcriteria for three sustainability pillars, namely economic, environmental, and social performance. Despite the fact that many articles on SSS and evaluation were published during COVID-19, there seems to be little research on sustainability issues to date. The goal of this study is to suggest a fuzzy multicriteria approach to SSCM planning. Additionally, using the TBL method, the problem of determining a current model for SSS in the supply chain was investigated. The linguistic value of the subjective preference of experts is represented by triangular fuzzy numbers. Fuzzy TOPSIS (technique for order preference by similarity to ideal solution) is proposed to use standard weights to rank SSS for qualitative performance evaluation. COVID-19, on the other hand, has a detrimental impact on SSS and company results. The organization’s performance suffers as a result of the COVID-19 shutdown. The proposed method is demonstrated using an example.

## Graphical Abstract

The supply chain cycle in detail.

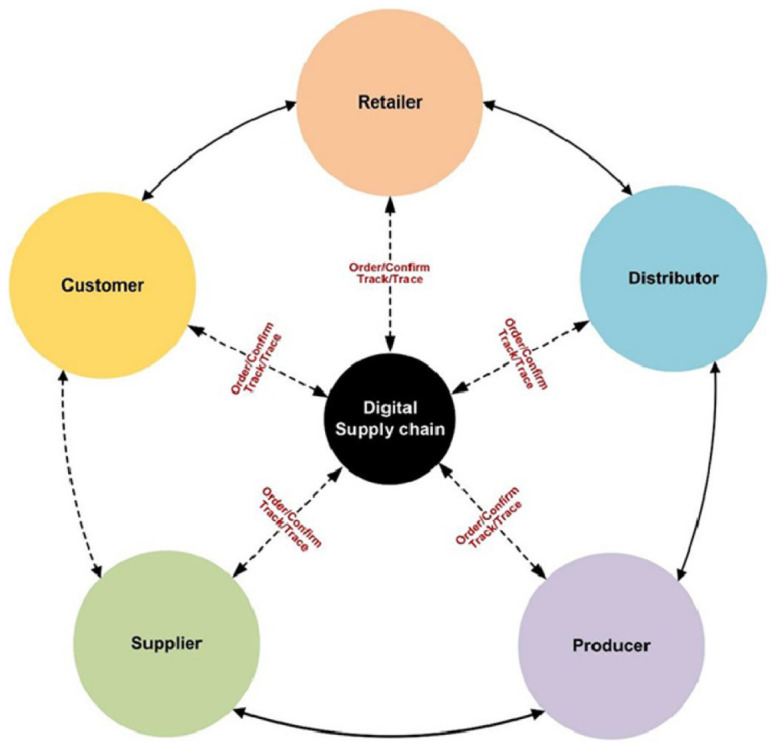

## Introduction

Over a period of time, significant changes have taken place in perceptions of enhancing the social and environmental performance of organizations. In the past few periods, due to the rapid natural resource consumption, concerns about the gap between the rich and the poor, and social responsibility, sustainability has become an important practice in professional research.

This concern has been demonstrated in legislation to expand organizational responsibilities, while increasing emphasis on the training of sustainable managers and in theories development to support sustainable supply chain management (SSCM) decisions (Zaid et al., 2018). In SCM, the sustainable supplier selection (SSS) is the main problem faced by managers in maintaining the strategic competitive position of the organization. From the first purchase of the product to the service provider at the end of its service life, the SSS can be applied to various SSS throughout the product life cycle (Hou et al., 2020). As the research literature proves, it is always necessary to consider the tangible and intangible factors of SSS assessment, and the definition of these factors is not always clear (Mani et al., 2018).

Usually, at the time of evaluating SSS the organizations consider cost, delivery, quality, price, technology, and flexibility criteria. Nowadays, logistics SCM solutions act as major factors in ensuring the competitiveness of the SCM, and the procurement actions have become more complicated due to three pillars of sustainability pressures. But now, thanks to many organizations to consider the adoption of a SSCM plan for economic, environmental, and social issues and to evaluate the sustainability performance of their SSS (Önüt et al., 2009). However, there are several evaluation models in the literature for SSS. There are many methods for reviewing SSS, such as mixed integer programming, sustainability triple bottom line (TBL) criteria (Hou et al., 2021), weighted linear model method, fuzzy sustainable manufacturing company index (FSMCI), analytic hierarchy process and linear programming model, human judgment model, neural network/case-based reasoning method, statistical analysis, etc. Most of the methods mentioned are based on the multiple properties of SSS (Li et al., 2007).

A number of studies have been carried out on green SSS. Yeh and Chuang (2011) studied a large number of papers on how to apply green principles by environmental principles, which was evaluated with the help of multiobjective strategic planning. Hung (2011) discusses a fuzzy objective planning method for green supply chain management (GSCM) optimization based on activity cost accounting and value chain performance evaluation. You et al. (2020) suggested that supplier selection and environmental performance was measured using a mixed fuzzy multicriteria strategic planning method. Govindan et al. (2020) integrated hilton supply management (HSM) into GSCM, and proposed a SSS model based on HSM using the ANP method. Liu et al. (2019) proposed a fuzzy TOPSIS method to evaluate the environmental performance of SSS. Govindan et al. (2013) reviewed a new fuzzy multicriteria strategic framework, which is used for SSS with the incomplete information services. More and more authors have solved SSS problems based on environmental aspects. The dual focus on environmental and economic standards in SSS must be further expanded to take in social sustainable criteria such as child labor, worker health, and social equity. Though these studies provide insights into the literature on sustainable/green SSS assessments, few people pay attention to SSS assessments that take all sustainability criteria into account. The main contributions of this paper include the SSS decision model in the SSCM based on the concept of TBL. Nicoletti et al. (2018) have emphasized that the differences between the social, environmental, and economic sustainability aspects have been absorbed.

**FIGURE 1:**
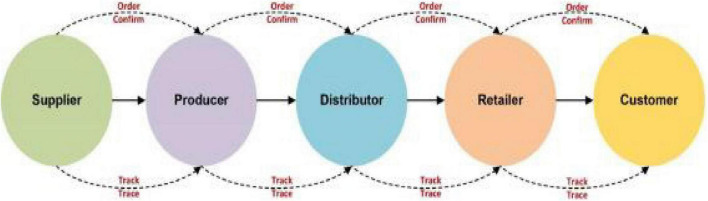
Traditional supply chain processes from supplier to customer.

In view of the past concerns and the multicriteria nature of SSCM issues, we have proposed a multicriteria framework to assess the sustainability performance of SSS. Multicriteria strategic methods in real-world systems usually deal with personal human preferences. Since human judgments and preferences are usually vague and complicated, experts cannot use accurate scale language assessments to estimate their preferences, but can only give accurate language valuations. However, the COVID-19 crisis has a negative impact on businesses. COVID-19 has a damaging effect on all organizations, demonstrating a negative impact on firm performance and SSS. Due to the lockdown, the supplier’s performance is rapidly deteriorating. As a result, the lockdown issue is causing the firms’ performance to deteriorate. There for firm’s performance increase, which aims to resolve such uncertainties (Hashemi et al., 2016). According to Iqbal S. et al. (2021), Iqbal W. et al. (2021) supply chain is a linear process on the movement of products. Indeed, in a supply chain, sellers offer raw materials to producers, producers ship finished products to distributors, distributors pass on specified quantity of goods to retailers, and retailers, in turn, sell the products to consumers (Hou et al., 2021). Figure 1 shows the whole supply chain processes from supplier to customer.

The following is the structure of this paper: First, principles of SSS by reviewing GSCM, social duty, and determining sustainability standards that affect the company’s purchasing decisions are reviewed (Iqbal et al., 2020). Second, to assess SSS’s environmental performance, the fundamentals of fuzzy set theory and the fuzzy TOPSIS approach are explained. Then, a method is applied and proposed numerically to perform sensitivity analysis on the results. Finally, a discussion and some concluding remarks are provided.

## Review of Literature

One of the most difficult aspects of sustainable development is to put the definition of the World Commission on Environment and Development into practice and guide decision-making with its terms of reference. Another method to describe sustainability is to assist in the design of human and industrial systems so that the use of natural resources and human recycling do not lead to a significant reduction in quality of life owing to bad conditions and the loss of future economic prospects, and the impact of social situations on human health and the atmospheree. So this definition clearly shows that concert indicators are required to judge the sustainability and success of any decision (Veleva et al., 2001).

The three pillars of sustainability and SSCM practice include a series of strategies. Although much work on green supply chain management (GSCM) has been done in the past, there is very little research on SSCM practices. To meet the needs of various stakeholder groups, increasing market pressure and stricter SSCM practices, organizations have begun to focus on their supply chains. Today, GSCM has become an important concern for companies that incorporate the three pillars of sustainability into their strategy (Rebitzer et al., 2004). The organization understands the necessity of partners taking responsibility for their own expansion’s long-term sustainability, and without SSCM practices it is impossible to solve the sustainability problems of any organization (Roy et al., 2020). Zafar et al. (2019) concluded that quality delivery and performance history of SSS in Pakistan has the most essential criteria, but COVID-19’s role has remained the most damaging to the global economy. The COVID-19 spread is causing the business to fall day by day. Markets are constricting and business revenue is shrinking. In the backdrop of COVID-19, most countries have stopped operations and ordered citizens to remain in their homes. One of the most important safeguards against COVID-19 is social isolation.

Sustainable supply chain management is defined as the management of materials and information flow, as well as the collaboration between companies in the supply chain. It also integrates the TBL selection issues including all three pillars of sustainable change (de Haan-Hoek et al., 2020). According to the TBL technique, firms must engage in activities that improve SSCM practizes and business performance in addition to their economic success (Khokhar et al., 2020b). By adopting a TBL approach, organizations assume a responsible position with regard to economic, environmental, social prosperity, quality, and justice, respectively (Rashidi et al., 2020).

There are many activities that can be incorporated into GSCM plans and SSCM practices (Ali et al., 2020; Yu et al., 2020). SSS and environmental cooperation includes activities aimed at improving environmental performance and SSS capabilities to carry out joint projects to develop green products and innovation (Li et al., 2020; Zhang et al., 2020b). SSS in GSCM is clearly a key activity in procurement management because the company’s SSS can prove the company’s environmental sustainability and ecological performance (Roehrich et al., 2017). The literature focusing on GSCM aims to obtain certification or introduce green practices through the three pillars of sustainability, so as to promote the SSS and improve SSCM’s practices and business preferences (Chiou et al., 2011).

### Selection Criteria for Social Supply Chain Management

Organizations are liable for social interests, and social interests can also be found in the company’s mission and value statement (Diers-Lawson et al., 2020). Although social duty has a long history, the concept of social duty (and sustainability) in the supply chain has only appeared in recent years (Osei-Kojo and Andrews, 2020). To implement the social responsibility system, stakeholders, consumer non-governmental organizations (NGOs), and local community regulations have put increasing pressure on the organization. In the SCM, these systems are used to transfer social responsible behaviors, especially those that affect their business partners, and provide benchmarks for environmental principles that the society must meet (Mani et al., 2020; Shafiq et al., 2020).

Social duty can be defined as the voluntary combination of environmental and social issues in the organization’s business operations and relationships with stakeholders (Halim Perdana Kusuma et al., 2019; Zhang et al., 2020a). Organizations are increasingly aware that their behavior in procurement and SSCM will greatly regard their status and long-standing success (Baloch et al., 2020). Administrations are responsible for environmental health and safety regulations that promote and protect workers who produce their products, whether they are direct employees or working for SSS (Testa et al., 2020).

**FIGURE 2:**
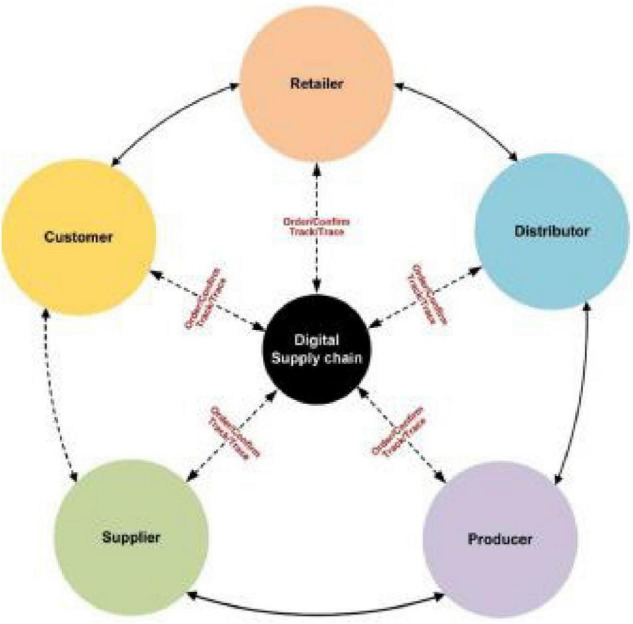
Membership function of triangular fuzzy number A.

Social duty has been the subject of many studies. Kelley et al. (2019) and Papacharalampous et al. (2019) believe that social responsibility includes the economic legal ethics and charity expectations imposed by the society on the organization at a specific time. Teh et al. (2019) and Zahid et al. (2020) follow categories as important aspects of ethical diversity working conditions at the social level, human rights, security, philanthropy, and communities. The SMEs encountered practices and difficulties in transferring social responsibility behavior to SSS doing business in developing countries, and also firm performance was affected by lockdown (Morsing and Spence, 2019; Zhang et al., 2019; Yumei et al., 2021). Oliveira et al. (2019) and Yazdani et al. (2019) have developed a framework for modeling and analyzing complicated universal SCM networks with undertaking social obligation through a comprehensive risk management and environmental decision-making. Many methods of presenting social responsibility and SSCM issues to reverse logistics systems have been studied (Sarkis et al., 2010; Nikolaou et al., 2013; Pakistan Bureau of Statistics, 2014). Figure 2 shows the membership function of triangular fuzzy number A.

### Sustainable Supplier Selection Criteria and Methods

**FIGURE 3:**
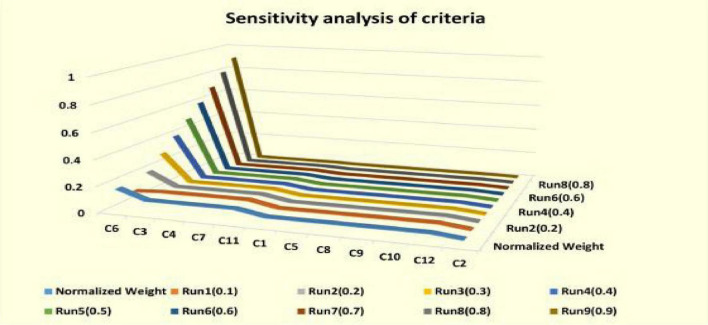
Sensitivity analysis.

The establishment of a standards system is one of the most significant operations in the process of making SSS judgments. Many scholars have been working on the development of these criteria since the 1960s. Zafar et al. (2019) were one of the first researchers in this field. The questionnaire papers were sent to the administrators of Pakistani companies and he determined 33 different SSS criteria (Khokhar, 2019). These standards include product quality, performance assurance, delivery and claims policy production facilities as well as production capacity net prices and technical capabilities. Zafar et al. (2019) concluded that quality delivery and performance history of SSS in Pakistan are the three most important criteria, but the role of current suppliers has remained most harmful to the world economy. Ikram et al. (2019) studied that the most important criteria for SSS are product quality, delivery, and performance in the past history of Pakistan. Muhammad et al. (2020) proposed the MCDM methods for SSS. They collected and analyzed relevant articles that appeared in international journals from 2001 to 2010 to solve the most important criteria considered by experts for SSS. Dweiri et al. (2016) summarized that since 1960s, many researchers have focused on the establishment of these economic criteria. Figure 3 shows the hierarchical structure of decision problem.

The development of social and green SSS is also essential for effective SSCM, and consideration of environmental and social factors must go beyond the forefront of the organization’s SSS agenda, but lockdown has a negative influence on the firm performance (Ageron et al., 2012; Khokhar et al., 2020a). The organization has adopted various methods and activities of SSS decisions is establishing the criteria. In this study, we concluded that some criteria can be applied in the SSS which defined them precisely (Hashemi et al., 2015). The selection criteria are not intended to fully describe the SSS performance, but only as an example of measures that can be formulated. We have summarized many standards and trials that can be measured in the literature in Table 1 from the regard of sustainability.

**TABLE 1:** Types of fuzzy model method and authors.

Methods	Type	Authors
Fuzzy TOPSIS, goal programming	Group model	
FVIKOR	Single model	
FVIKOR	Single model	
FMLMCDM, FTOPSIS, and FMOORA	Group model	
FAHP, ARASF, and MSGP	Group mode	
IT2 FSs-based TODIM	Group mode	Khokhar, 2019
BWM and fuzzy TOPSIS	Group mode	
Fuzzy set, TODIM, PROMETHEE, Fuzzy-TOPSIS, Fuzzy-VIKOR	Group mode	
Fuzzy AHP and Fuzzy MOORA	Group mode	
BWM, Fuzzy TOPSIS, and FMOLP	Group mode	
Fuzzy AHP-TOPSIS	Group mode	
Fuzzy MOORA and FMEA	Group mode	Iqbal et al., 2019a
Fuzzy MADM, TBL, QFD, and Fuzzy VIKOR	Group mode	
ANN, FAHP, and FTOPSIS	Group mode	
AHP Sort II, Interval type-2 fuzzy sets	Group mode	Iqbal et al., 2019b
Fuzzy VIKOR	Single model	
Rough-fuzzy DEMATEL-TOPSIS	Group model	
Spherical fuzzy AHP	Single model	
Fuzzy SWARA and Fuzzy ARAS	Group model	
Fuzzy multi-objective optimization fuzzy goal programming	Group model	Irshad et al., 2019
Fuzzy linear programming	Single model	
Fuzzy data envelopment analysis	Single model	
Fuzzy neural networks	Single model	
Clustering method	Single model	

## Fuzzy Numbers

Natural language expressing awareness or judgment is always personal, unclear, or imprecise. The uncertainty and subjectivity of fuzzy numbers have been dealt with by probability and statistics for a long time. Since the accuracy of words is not as good as numbers, the concept of linguistic variables generally describes the definition of events (Chien et al., 2021). The definitions of these events are too poor to be described in predictable quantitative terms. To solve the perspicacity of human intelligence, Chou et al. (2008) introduced fuzzy set theory to precise the linguistic specifications in the process of experts. Fuzzy theory enables experts to deal with the ambiguity involved in data language evaluation. Wang and Lin (2003) were the first researchers to use fuzzy sets to investigate decision-making problems and initiated the FMCDM method. This article uses triangular fuzzy numbers to evaluate experts’ preferences (Fu et al., 2021). The purpose for using triangular fuzzy numbers is that experts are instinctively easy to use and calculate.

There are various ways to define fuzzy numbers. A is a real fuzzy number which is described as fuzzy subset of the real line *R* with membership function fA(x), it is a constant mapping from x in X to the closed interval [0, 1]. If the membership level of an element is 1, it means that the element must be in the set. If the member level is 0, it means that the element is definitely not in the set. This article defines the perception of fuzzy numbers as follows (Enginoglu et al., 2011).

**Definition 1.** The fuzzy number’s membership functions is shown in Figure 1:


(1) $$f_{A}\,\left(x\right)=\left\{ \begin{array}{cc} 0 & x\,\left\langle a_{;}\,x\right\rangle \,c\\ \frac{x-a}{b-a}, & a\leq x\leq b\\ \frac{c-x}{c-b}, & b\leq x\leq c \end{array}\right.$$

**Definition 2.** Let A = (a, b, c) and B = (a_1_, b_1_, c_1_) be two triangular fuzzy numbers. Then the operational laws of these two triangular fuzzy numbers are as follows:


(2) $$A\,\left(+\right)\,B=\left(a,b,c\right)\,\left(+\right)\,\left(a\,1,b\,1,c\,1\right)=\left(a+a_{1},b+b_{1},c+c_{1}\right)$$

(3) $$A\,\left(-\right)\,B=\left(a,b,c\right)\,\left(-\right)\,\left(a\,1,b\,1\,c\,1\right)=\left(a-a_{1},b-b_{1},c-c_{1}\right)$$

(4) $$A{(}^{*})B=\left(a_{;}b_{;}c\right){(}^{*})\left(a1,b1,c1\right)=\left(a^{*}a_{1},b^{*}b_{1},c^{*}c_{1}\right)$$

(5) $$\left(A\,\left(/\right)\,B=\left(a,b,c\right)\,\left(:\right)\,\left(a\,1,b\,1,c\,1\right)=\left(a/a\,1\,b/b_{1}\,c/c_{1}\right)\right)$$

(6) $$K^{*}\,A=\left(k^{*}\,a_{,}\,k^{*}\,b_{,}\,k^{*}\,c\right)$$

(7) (A)-1=(1/c,1/b,1/a)

The distance between A, B fuzzy numbers is calculated as:


(8) $$d\,\left(A_{;}\,B\right)=\sqrt{1/3\,\left[{\left(a-a_{1}\right)}^{2}\,{\left(b-b_{1}\right)}^{2}\,{\left(c-c_{1}\right)}^{2}\,\right]}$$

**Definition 3.** Assume that an expert group has K expert, and the fuzzy rating of each expert (*k* = 1, 2,..,*K*) can be represented as a positive triangular fuzzy number *R_k_*(*k* = 1, 2,..,*K*) with membership function*f*_*Rk*_(*x*). Then, the aggregated fuzzy number is defined as:


(9) R=(a,b,c),k=1,2,..,K

where *a* = min*_k_*{*a*_*k*_},*b* = 1/*k* $\sum_{k}^{k}$ = 1*b*_*k*_,*c* = max*_k_*{*c*_*k*_}

## The Fuzzy Topsis Method

**FIGURE 4:**
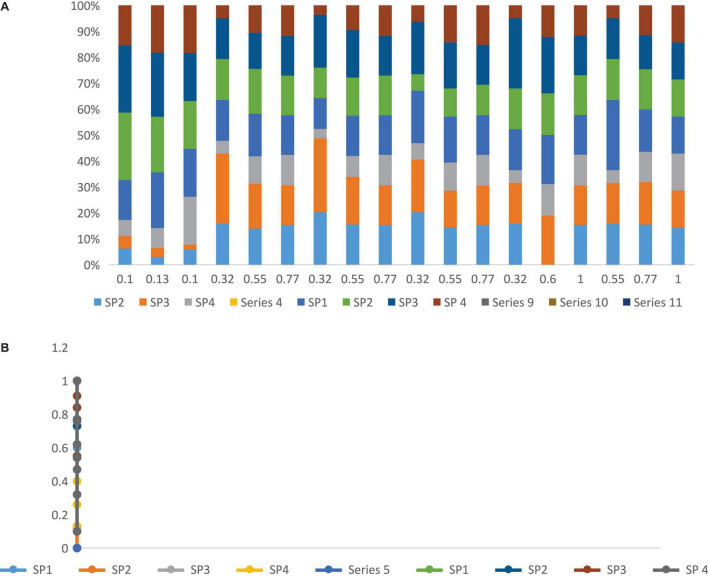
**(A)** Show the weighted normalized fuzzy decision matrix. **(B)** Show the weighted normalized fuzzy decision matrix.

The multiattribute decision-making (MADM) technology functionally related to the problem of discrete alternatives is a practical tool for resolving real-world problems. Since many MADM technologies are involved, Gati et al. (1996) provide taxonomies to classify these technologies into information types from experts, prominent information features, and main method categories. Classification does provide us with a clear direction for learning MADM technology (Bernroider and Stix, 2006). In these technologies, since there is a clearly expressed process, the attribute information category from the experts with the information is convenient for decision-making. Table 2 and Figures 4A,B show the weighted normalized fuzzy decision matrix. In this category of TOPSIS, the distance measurement concept as an alternative to the positive ideal solution (PIS) and negative ideal solution (NIS) is the most direct technique in MADM. Table 3 describes the distances between suppliers (SP) and A*, A with respect to each criterion.

**TABLE 2:** Weighted normalized fuzzy decision matrix.

Experts	C11		C12		C13		C14		C21		C22	
SP1	0.05	0.1	0.16	0.6	0.16	0.6	0.9	0.53	0.16	0.8	0.27	0.8
SP2	0.006	0.14	0.16	0.8	0.27	0.8	0.9	0.6	0.16	0.8	0.16	0.8
SP3	0.05	0.9	0.27	0.8	0.38	0.8	0.9	0.6	0.16	0.8	0.16	0.8
SP4	0.06	0.2	0.05	0.6	0.05	0.6	0.02	0.53	0.05	0.6	0.005	

	**C23**		**C24**		**C31**		**C32**		**C33**		**C34**	

SP1	0.16	0.51	0.9	0.6	0.16	0.8	0.16	0.8	0.9	0.8	0.16	0.9
SP2	0.27	0.51	0.9	0.6	0.16	0.8	0.05	0.7	0.9	0.8	0.9	0.8
SP3	0.27	0.59	0.9	0.6	0.28	0.8	0.16	0.8	0.16	0.8	0.9	0.9
SP4	0.16	0.43	0.002	0.53	0.05	0.6	0.005	0.8	0.02	0.6	0.02	0.8

**TABLE 3:** Distances between suppliers (SP) and A*, A with respect to each criterion.

	C11	C12	C13	C14	C21	C22	C23	C24	C31	C32	C33	C34
d (SP1, A*)	0.52	0.51	0.42	0.45	0.39	0.78	0.47	0.37	0.46	0.46	0.53	0.49
d (SP2, A*)	0.49	0.39	0.4	0.44	0.45	0.78	0.39	0.39	0.37	0.46	0.48	0.55
d (SP3, A*)	0.4	0.3	0.4	0.44	0.45	0.78	0.39	0.39	0.37	0.46	0.48	0.55
d (SP 4, A*)	0.59	0.59	0.47	0.57	0.55	0.61	0.49	0.46	0.58	0.54	0.62	0.59
d (SP1, A^–^)	0.41	0.42	0.32	0.57	0.14	0.46	0.44	0.57	0.56	0.4	0.53	0.54
d (SP 2, A^–^)	0.53	0.58	0.41	0.55	0.57	0.14	0.46	0.44	0.56	0.4	0.53	0.54
d (SP3, A^–^)	0.57	0.64	0.41	0.58	0.57	0.07	0.49	0.42	0.62	0.56	0.56	0.52
d (SP 4, A^–^)	0.4	0.39	0.31	0.41	0.52	0.49	0.44	0.32	0.4	0.53	0.4	0.51

Meanwhile, this study proposes related technologies, such as ELECTRE and AHP, and the characteristics of the TOPSIS method make it a major MADM technology (Kahraman et al., 2007; Kalbar et al., 2012):

• First and for most take unlimited range of all three pillars of sustainability performance attributes and criteria.
• Then clear trade-offs and interactions between performance attributes. More precisely, the change of any one attribute can be compensated by other attributes in an opposite or direct way.
• The MADM technology (such as ELECTRE) method only determines the level of each alternative, and the priority ranking of alternatives with numerical values can better understand the differences and similarities between alternatives (Hou et al., 2019).
• AHP methods circumvents the pair-wise evaluation. This method is used when dealing with a large number of sustainability criteria/subcriteria.
• This is systematic simple calculation process.
• In general simulation comparison, when adding or deleting alternative methods in the MADM method, the rank inversion of TOPSIS is the smallest.
• The TOPSIS solution method includes the following steps (Opricovic and Tzeng, 2004; Yue, 2011; Memari et al., 2019):

**Step 1**. To compute the normalized decision matrix. The normalized fuzzy-decision matrix can be expressed as:


$$R={\left[r_{i\,j}\right]}_{m+n}$$

where B and C are the sets of product cost criteria and benefit correspondingly:


$$r_{i\,j}=\left(\frac{a_{i\,j}}{c_{j}^{*}},\frac{b_{i\,j}}{c_{j}^{*}},\frac{c_{i\,j}}{c_{j}^{*}}\right),j\in B$$

(10) $$c_{j}^{*}={\text{max}}_{i}\,c_{ij,}\,j\in B$$

$$r_{i\,j}=\left(\frac{a_{j}^{-}}{c_{i\,j}},\frac{a_{j}^{-}}{b_{i\,j}},\frac{a_{j}^{-}}{a_{i\,j}}\right),j\in C$$

(11) $$a_{j}^{-}={\text{min}}_{i}\,a_{ij,}\,j\in C$$

The above normalization method aims to retain the standardized attributes of the element *r*_*ij*_ (normalized) triangular fuzzy number.

**Step 2.** To estimate the weighted normalized decision matrix. The weighted normalized value *v*_*ij*_ is considered as:


(12) $$V={\left[v_{i\,j}\right]}_{m^{*}\,n}\,i=1_{,}\,2_{,}\,\ldots ,m\,j=1_{,}\,2_{,}\,\ldots ,n$$

where *v*_*ij*_ = *r*_*ij*_.*w*_*ij*_ and *w*_*ij*_ are the weights of the jth attribute, or standard.

**Step 3**. To determine positive and negative ideal solutions: Fuzzy positive ideal solution (FPIS, A*) and fuzzy negative ideal of the solution (FNIS, *A*^−^) can be defined as:


(13) $$A^{*}=\left(v_{1,}^{*}\,v_{1}^{*},\ldots ,v_{n}^{*}\right)$$

(14) $$A^{-}=\left(v_{1}^{-},v_{2}^{-},\ldots ,v_{n}^{-}\right)$$

where $\nu_{j}^{*}={\text{max}}_{i}\,\left\{v_{i\,j\,3}\right\}$ and $v_{j}^{-}={\text{min}}_{i}\,{\left\{\nu_{i\,j\,1}\right\}}_{;}\,i=1$; 2; *m*_;_*j* = _1;_2_;_ *n*

**Step 4**. The distance of each alternative from the positive and the negative ideal solution A*,*A*^−^ can be calculated as:

$\text{where}\,\nu_{j}^{*}={\text{max}}_{i}\,\left\{v_{j\,j\,3}\right\}$ and $\nu_{j}^{-}={\text{min}}_{\text{i}}\,\left\{\nu_{i\,j\,1}\right\},i=1,2,\ldots ,m,$*j* = 1,…2,, *n*


(15) $$d_{i}^{*}=\sum_{j=1}^{n}d_{\nu }\,\left(\nu_{ij,}\,\nu_{j}^{*}\right),i=1,2,\ldots ,m$$

(16) $$d_{i}^{-}=\sum_{j=1}^{n}d_{\nu }\,\left(\nu_{ij,}\,\nu_{j}^{-}\right),i=1,2,\ldots ,m$$

and *d*_*v*_(0,0) is the distance measurement among two fuzzy numbers.

**Step 5.** To estimate the virtual proximity to the ideal resolution. One defines the tightness factor to determine all ranking orders possible SP after *d_i* and $d_{i}^{-}$ of each alternative *A*_*i*_(*i* = 1, 2,.,*m*) has been calculated. For the closeness coefficient (CC_l_), the alternative calculation is:


(17) $${\text{CC}}_{l}=d_{i}^{-}/\left(d_{i}+d_{i}^{-}\right),i=1,2,\ldots ,m$$

**Step 6**. To arrange the order of preferences. Alternative Ai is closer when criteria change index (cci) approaches 1, FPIS (A*) moves away from FNIS (*A*^−^). According to the descending order of cci, we can determine sort all alternatives and choose one of the best possible alternatives.

## Illustrative Case and Results

**FIGURE 5:**
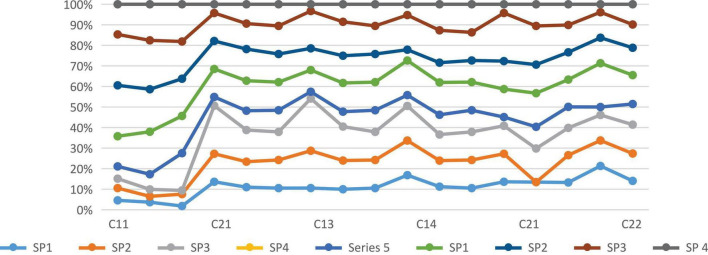
Fuzzy TOPSIS results and sensitivity analysis of sustainable supplier (SP) selection.

To test the practicability of the proposed SSS and evaluation methods, a case of evaluation is illustrated. Figure 2 shows the hierarchy of the conclusion problem. We present the main criticisms identified in Table 1. We conducted surveys by distributing questionnaires to managers in the areas of business purchase and environment. The assessment consequences determined the comparative significance weights of several standards and grades. As described in Table 1 and Figure 3 there are four economic, environmental, and social criteria (C11, C12, C13, and C14), (C21, C22, C23, and C24), (C31, C32, C33, and C34), respectively. C11 is the product cost criteria. Figure 5 described the Fuzzy TOPSIS results and sensitivity analysis of sustainable supplier (SP) selection.

**FIGURE 6:**
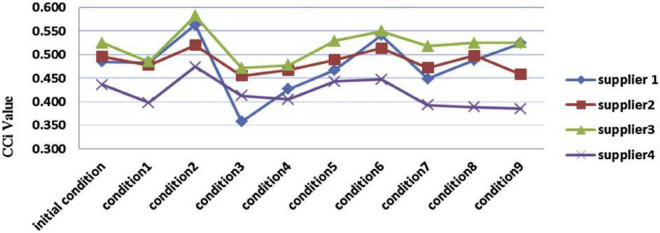
Sensitivity analysis result.

Thus the currently proposed method is used to solve this problem. Table 4 defines the relative importance weight and rank importance of the criteria described using linguistic variables. The three experts expressed their views on the importance weight of the 12 subcriteria of three pillars and the rating of each SSS relative to these criteria/subcriteria. Tables 5, 6 show the original evaluation information provided by the three experts. Tables 5–7 show the fuzzy decision matrix and fuzzy weights of the standard normalized fuzzy decision matrix for the distance of each SSS to FPIS and FNIS and the proximity coefficient of each SSS for each criterion, respectively. According to the SSS choice, we used Ms Excel to complete all calculations. Figure 6 shows the sensitivity analysis result.

**TABLE 4:** Linguistic variables.

Linguistic variables for the fuzzy numbers		
**Linguistic variables**	**Code**	**Fuzzy scale**
Very good	VG	(7,9)
Good	G	(5,9)
Fair	F	(5,7)
Poor	P	(1,3)
Very poor	VP	(1,3)
Very high	VH	(0.7,0.9)
High	H	(0.5,0.9)
Medium	M	(0.3,0.5)
Low	L	(0.1,0.5)
Very low	VL	(0.1,0.3)

**TABLE 5:** The importance of the three weighting criteria from experts.

Economic criteria					Environmental criteria				Social criteria			
Experts	C11	C12	C13	C14	C21	C22	C23	C24	C31	C32	C33	C34
Expert1	M	H	VH	M	VH	H	H	M	M	H	M	H
Expert2	VH	M	H	H	H	VH	M	VH	H	M	H	M
Expert3	H	H	VH	M	VH	H	H	M	VH	H	VH	H

**TABLE 6:** Evaluation of suppliers (SP) on sustainability criteria by experts.

		Economic criteria				Environmental criteria				Social criteria			
Experts	Suppliers	C11	C12	C13	C14	C21	C22	C23	C24	C31	C32	C33	C34
Expert1	SP1	G	F	F	F	F	G	G	F	G	F	F	G
	SP2	F	F	G	F	F	G	G	VG	G	F	G	F
	SP3	VG	G	VG	G	F	F	G	G	VG	VG	G	F
	SP4	P	F	F	P	P	F	F	F	P	P	F	F
Expert2	SP1	G	F	F	F	G	G	G	F	F	VG	G	G
	SP2	F	G	G	F	F	F	G	G	G	F	F	G
	SP3	G	G	VG	F	G	G	VG	F	VG	F	G	G
	SP4	F	P	P	F	F	G	F	P	F	G	P	P
Expert3	SP1	G	F	F	F	G	G	F	VG	VG	G	G	G
	SP2	F	F	G	G	G	G	F	F	F	P	F	G
	SP3	G	G	G	F	G	VG	G	F	G	G	VG	F
	SP4	P	F	P	F	M	P	G	F	F	P	F	G

**TABLE 7:** Fuzzy set decision matrix and fuzzy weight of criteria.

Experts	C11	C12	C13	C14	C21	C22	C23	C24	C31	C32	C33	C34
Weight	0.4	0.4	0.4	0.2	0.5	0.4	0.4	0.2	0.4	0.2	0.2	0.2
SP1	4	2	2	2	2	5	2	2	2	2	2	4
SP2	2	2	4	2	2	2	4	2	2	1	2	2
SP3	4	4	6	2	2	2	4	2	4	2	4	2
SP4	1	1	1	1	1	1	2	1	1	1	1	1

**FIGURE 7:**
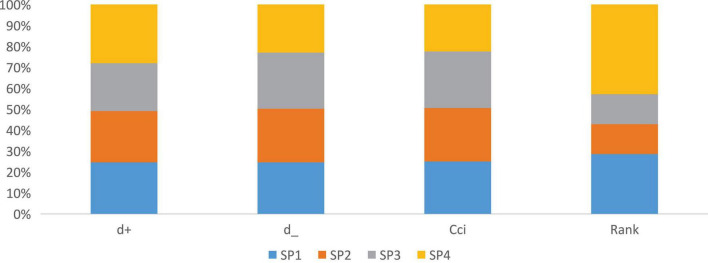
Calculations of d+, d–, and criteria change index (cci) from the Eq. 15 till Eq. 17.

Table 8 and Figure 7 summarize the final results of fuzzy TOPSIS analysis. According to the value of proximity coefficient (CCl), the rank order of the four SSS according to their sustainability performance is: SSS 2 > SSS 3 > SSS 4 > SSS 1. Therefore, from the perspective of experts, we can conclude that SSS 2 has the best sustainability performance. After considering all sustainability criteria, we have just shown the results of our analysis of SSS. After considering all sustainability criteria, we have just shown the results of our analysis of SSS.

**TABLE 8:** Calculations of d^+^, d^–^, and cci giving to Eq. 15 till Eq. 17.

	d^+^	d^_^	Cci	Rank
SP1	5.94	5.61	0.485	2
SP2	5.92	5.83	0.495	1
SP3	5.52	6.11	0.524	1
SP4	6.77	5.23	0.435	3

The purpose of the sensitivity analysis is to deliberate the impact on SSS position when we select altered experts and criteria. This query is useful when there is uncertainty in the definition of the importance of different factors. Table 9 and Figure 3 give detailed information on the other nine conditions. According to this sensitivity analysis, changing the fuzzy weight will change the order of SSS. Although the ranking of SSS varies depending on the basis of weights, usually from the all SSS, the SSS 2 is the best. Since the decision-making process is a sensitive type of criteria, the expertise should be carefully considered when choosing this process. As in Table 10 the fuzzy topsis method show the result of sensitivity analysis to sustainable supplier (SP) selection.

**TABLE 9:** Fuzzy TOPSIS method is the result of sensitivity analysis to sustainable supplier (SP) selection.

Condition	Decision criteria	Experts	Suppliers (SP) ranking (Respectively)
Initial condition	C11, C12, C13, C14, C21, C22, C23, C24, C31, C32, C33, C34	Expert1, Expert2, Expert3	SP2, SP3, SP4, SP1
Condition1	C21, C22, C23, C24	Expert1, Expert2, Expert3	SP2, SP3, SP4, SP1
Condition2	C31, C32, C33, C34	Expert1, Expert2, Expert3	SP2, SP3, SP4, SP1
Condition3	C11, C21, C13, C14	Expert1, Expert2, Expert3	SP2, SP3, SP4, SP1
Condition4	C11, C21, C13, C14, C21, C22, C23, C24	Expert1, Expert2, Expert3	SP2, SP3, SP4, SP1
Condition5	C11, C21, C13, C14, C31, C32, C33, C34	Expert1, Expert2, Expert3	SP2, SP3, SP4, SP1
Condition6	C21, C22, C23, C24, C31, C32, C33, C34	Expert1, Expert2, Expert3	SP2, SP3, SP4, SP1
Condition7	C11, C21, C13, C14, C21, C22, C23, C24, C31, C32, C33, C34	Expert1	SP2, SP3, SP4, SP1
Condition8	C11, C21, C13, C14, C21, C22, C23, C24, C31, C32, C33, C34	Expert2	SP2, SP2, SP4, SP1
Condition9	C11, C12, C13, C14, C21, C22, C23, C24, C31, C32, C33, C34	Expert3	SP2, SP3, SP1, SP1

**TABLE 10:** Normalized fuzzy decision matrix.

Experts	C11	C12	C13	C14	C21	C22	C23	C24	C31	C32	C33	C34
SP1	0.1	0.13	0.1	0.32	0.55	0.77	0.32	0.55	0.77	0.32	0.55	0.77
SP2	0.13	0.1	0.32	0.32	0.62	1	0.55	0.77	1	0.32	0.62	1
SP3	0.1	0.12	0.1	0.55	0.77	1	0.77	0.91	1	0.32	0.62	1
SP4	0.13	0.26	1	0.1	0.47	0.77	0.1	0.4	0.77	0.1	0.47	0.771

## Conclusion and Future Research Directions

Sustainable supply selection, the environment, and social cooperation are all supply chain management edges that can help achieve TBL benefits and promote long-term social development. Based on the TBL concept, this article focuses on the economic, environmental, and social criteria of SSS. All three sustainability features should be studied simultaneously in a comprehensive study of sustainable supply chain operations. In this paper, we present a fuzzy MCDM method for SSS decisions that is based on sustainability criteria. First, based on the literature, the SSS criteria was determined. Second, experts used fuzzy TOPSIS to aggregate scores and generate overall performance scores to assess SSCM practizes and business performance. Finally, we performed a sensitivity analysis to determine the decision-making process’ standard weights. The findings advised the company to choose the best SSS among the candidates in four ways to continue working with the SSS team, as well as suggesting that certain SSS improve certain defects or stop working with certain SSS. In general, one of the most important factors is the selection of SSS. This, too, is based on expert judgement. Opportunities for improving the company’s sustainability performance can be discovered and prioritized through its decision-making and implementation, reducing the activity’s negative impact on the environment and society.

However, there are a few limitations to the above-mentioned article. There are over ten SSCM practizes that have been identified. Since no other SSCM practizes or issues have been discovered, actual concerns about the accuracy of these experts’ decision-making must be investigated to ensure the method’s viability. As this study was conducted during a lockdown, the companies were not fully operational, which may have led to data collection flaws. As a result, future research should focus on other countries to examine the impact of green supply chain management and SSCM practizes on firm performance. One of the limitations of this operation’s feasibility is the amount of information and data required to apply this method. Not only should supply chain managers adopt this strategy, but they should also keep such data for future organizational management. Experts are under time constraints and lack expertise on issues related to GSCM and SSCM practizes as a result of the SSS evaluation process. Despite the fact that the preferences are not exhaustive, we recommend that you consider SSS. Perhaps this will be the subject of future research. Various technologies and dynamic evaluation models can also be used to combine the SSS phase with continuous examination. Furthermore, after positioning all SSS, demand allocation is an important issue that may become a new trend in the future. However, COVID-19 has a negative effect on company performance. Due to the use of COVID-19, countries are under lockdown and business operations seem to be paralyzed. Therefore, the lock-in situation due to COVID-19 has a negative impact on the company’s performance.

## Author Contributions

YH: conceptualization and data curation. MK: methodology, writing – original draft, data curation, visualization, and supervision. SZ: visualization and editing. AS: review and editing, review, editing, and software. All authors contributed to the article and approved the submitted version.

## Conflict of Interest

The authors declare that the research was conducted in the absence of any commercial or financial relationships that could be construed as a potential conflict of interest.

## Publisher’s Note

All claims expressed in this article are solely those of the authors and do not necessarily represent those of their affiliated organizations, or those of the publisher, the editors and the reviewers. Any product that may be evaluated in this article, or claim that may be made by its manufacturer, is not guaranteed or endorsed by the publisher.
